# Epidemiological Profile and Survival Outcomes of Laryngeal Cancer in Western Greece: A 21-Year Retrospective Cohort Study

**DOI:** 10.3390/jcm15082868

**Published:** 2026-04-09

**Authors:** Christos S. Avdulla, Nicholas Mastronikolis, Ntaniela Tachirai, Eleni Jelastopulu

**Affiliations:** 1 Department of Public Health, Epidemiology and Quality of Life, University of Patras, 26504 Patras, Greece; cavntoulla@upatras.gr; 2 Department of Otorhinolaryngology, University of Patras, 26504 Patras, Greece; nmastr@upatras.gr; 3 Infectious Diseases Unit, University General Hospital “Attikon”, 12462 Athens, Greece; eladaani@gmail.com

**Keywords:** laryngeal cancer, epidemiology, survival analysis, prognostic factors, retrospective cohort study, disease-specific survival (DSS), overall survival (OS), Western Greece

## Abstract

**Background/Objectives**: Laryngeal cancer remains a global health burden, particularly in regions with high tobacco and alcohol consumption. This study aimed to provide a comprehensive epidemiological overview of laryngeal cancer in Western Greece and to assess overall survival (OS), disease-specific survival (DSS), and key prognostic factors over a 21-year period. **Methods**: A retrospective cohort study was conducted, including patients diagnosed and treated for laryngeal cancer at the Otorhinolaryngology Department of the University General Hospital of Patras between 1997 and 2017. Demographic, clinical, histopathological, and treatment data were collected. Survival outcomes were estimated using Kaplan–Meier analysis and compared using the log-rank test. Multivariable Cox proportional hazards regression was performed to identify independent prognostic factors. Significance was set at *p* ≤ 0.05. **Results**: A total of 211 patients were included (mean age 62.7 years; 95.3% male). Active smoking was reported in 97.6% of cases. Most patients (88.6%) were diagnosed at advanced stages (III–IV), with glottic tumors being the most common (61.1%). The 5-year OS and DSS rates were 47.0% and 55.6%, respectively. Larger tumor size, nodal involvement, and advanced stage were significantly associated with reduced DSS in univariable analysis (*p* < 0.001). Cox regression confirmed tumor size (HR = 1.665, 95% CI: 1.187–2.336) and nodal status (HR = 1.546, 95% CI: 1.176–2.031) as independent predictors of DSS. **Conclusions**: The findings highlight the impact of advanced disease at diagnosis and the central prognostic role of tumor burden in laryngeal cancer in Western Greece. Early detection and timely management remain essential to improve patient outcomes.

## 1. Introduction

The larynx is a vital organ of the upper respiratory tract, contributing to respiration, airway protection during swallowing, and voice production. Its functional integrity is closely linked to communication and overall quality of life, making laryngeal disorders particularly impactful for affected individuals [1,2]. Laryngeal cancer represents one of the most common malignancies of the head and neck region and remains a significant global public health concern. The vast majority of tumors are squamous cell carcinomas (LSCC) and may arise in any of the laryngeal subsites, including the glottis, supraglottis, and subglottis [3].

The disease is strongly associated with well-established behavioral and environmental risk factors, particularly tobacco use and excessive alcohol consumption, which exert synergistic carcinogenic effects [4]. Additional contributors such as occupational exposure to carcinogens, poor oral hygiene, dietary habits, gastroesophageal reflux, and viral infections, including HPV, have also been implicated [5,6]. Tumor location influences symptom onset and, consequently, the stage at diagnosis. Glottic tumors often present early due to voice changes, whereas supraglottic tumors may remain silent until advanced stages, leading to delayed presentation and worse prognosis [3]. Treatment options, ranging from surgery and radiotherapy to chemotherapy or multimodal approaches, are guided by tumor stage, size, nodal involvement, and laryngeal subsite, all of which remain key determinants of survival [7].

Despite representing a relatively small proportion of all malignancies, laryngeal cancer remains an important component of head and neck cancers worldwide. According to GLOBOCAN 2022 estimates, approximately 189,000 new cases and more than 103,000 deaths from laryngeal cancer occur globally each year, highlighting the considerable global burden of the disease [8].

Across Europe, laryngeal cancer incidence has declined in many high-income countries in recent decades, largely reflecting reduced tobacco consumption. However, these trends are not uniform, and significant regional heterogeneity persists [9]. Despite advances in diagnostic and therapeutic strategies, overall survival has not improved substantially, partly due to the persistent predominance of late-stage diagnosis and the continued impact of modifiable risk factors, underscoring the need for earlier detection and optimized management pathways [10]. Considerable variation is also observed among European countries with regard to disease burden, access to care, and survival outcomes [9].

In Greece, both national and regional epidemiological data on laryngeal cancer are limited. Few studies have comprehensively evaluated demographic characteristics, clinical patterns, or long-term survival. Differences in healthcare accessibility, socioeconomic conditions, and diagnostic practices may contribute to regional disparities [11]. Western Greece, in particular, remains underrepresented in the literature, with a notable absence of long-term, population-based analyses capable of informing prevention strategies, clinical pathways, and resource allocation.

This study aimed to address this gap by providing a comprehensive epidemiological assessment of laryngeal cancer in Western Greece over a 21-year period. Specifically, it sought to describe patient demographic and clinical characteristics, estimate overall survival (OS) and disease-specific survival (DSS), and identify independent prognostic factors influencing outcomes. By delivering robust long-term data from a major tertiary referral center, this study contributes meaningful evidence to support early detection strategies, targeted prevention efforts, and improved oncologic care in the region.

## 2. Materials and Methods

### 2.1. Study Design and Setting

This retrospective cohort study was conducted at the Otorhinolaryngology Department of the University General Hospital of Patras, a major tertiary referral center in Western Greece. The study included patients diagnosed and treated for primary laryngeal cancer between January 1997 and December 2017.

### 2.2. Study Population

Eligible participants were adults with newly diagnosed, histologically confirmed primary laryngeal squamous cell carcinoma. No additional exclusion criteria were applied beyond the requirement for complete clinical, histopathological, and follow-up data; cases with insufficient or missing essential information were not included. All eligible patients were included in the overall survival (OS) analysis. For disease-specific survival (DSS), death from laryngeal cancer was defined as the event of interest, while patients who were alive at last follow-up or who died from causes unrelated to laryngeal cancer were treated as censored observations at the time of last follow-up or death.

### 2.3. Data Collection

Demographic, clinical, pathological, and treatment-related variables were extracted from medical records. Demographic data included age, sex, smoking status, and alcohol consumption. Clinical variables encompassed tumor location, AJCC TNM stage, nodal status, and follow-up outcomes. Histopathological information included tumor differentiation grade. Treatment data covered surgical management, radiotherapy, chemotherapy, and multimodal therapeutic approaches.

### 2.4. Definitions and Variable Classification

Tumor staging was based on the American Joint Committee on Cancer’s (AJCC) 7th edition. Smoking and alcohol consumption were recorded as demographic variables. OS was defined as the time from diagnosis to death from any cause or last follow-up. DSS was defined as the time from diagnosis to death specifically attributable to laryngeal cancer.

### 2.5. Outcomes

The primary outcomes were overall survival (OS) and disease-specific survival (DSS), assessed at 1, 3, 5, and 10 years after diagnosis. Secondary outcomes included the identification of prognostic factors influencing DSS. Variables evaluated as potential predictors included age, sex, smoking status, alcohol consumption, tumor histological characteristics, AJCC stage, anatomical subsite of the tumor, nodal involvement, and treatment modality.

### 2.6. Statistical Analysis

Descriptive statistics were used to summarize patient characteristics. Survival time was calculated from the date of initial surgical treatment to the date of death or last follow-up for both overall survival (OS) and disease-specific survival (DSS). Survival curves for OS and DSS were generated using the Kaplan–Meier method, and comparisons between groups were performed using the log-rank test. To further evaluate disease-specific mortality in the presence of competing events, a competing risks analysis was performed, treating death from causes unrelated to laryngeal cancer as a competing event. Cumulative incidence functions were estimated using the cmprsk package in R (version 4.5.3; R Foundation for Statistical Computing, Vienna, Austria). Multivariable Cox proportional hazards regression was used to identify independent prognostic factors. Multicollinearity among the independent variables included in the multivariable model was assessed using the variance inflation factor (VIF), with values greater than 5 considered indicative of potential collinearity. Analyses were performed using SPSS version 27 (IBM Corp., Armonk, NY, USA), and statistical significance was set at *p* ≤ 0.05.

### 2.7. Ethical Considerations

All ethical and regulatory requirements were strictly followed. Patient anonymity and data protection were ensured in accordance with the General Data Protection Regulation (GDPR). The study received approval from the Ethics, Research, and Deontology Committee of the University General Hospital of Patras (protocol number 187/4 May 2023). Due to the retrospective design, the requirement for informed consent was waived. The study adhered to the principles of the Declaration of Helsinki.

## 3. Results

### 3.1. Demographic Characteristics

A total of 211 patients were included in the study (Table 1). The majority were male (95.3%), whereas females represented only 4.7%. Most patients were active smokers (97.6%), with 47.9% reporting 51–100 pack-years and 29.9% reporting more than 100 pack-years. Heavy alcohol consumption was documented in 43.1% of cases, while 24.2% reported moderate intake. Patient age ranged from 29 to 89 years (mean 62.67 ± 11.43 years), while smoking exposure ranged from 12 to 240 pack-years (mean 88.15 ± 41.95 pack-years).

### 3.2. Tumor and Histopathological Characteristics

Tumor characteristics are summarized in Table 2. Nearly half of the patients presented with T4 tumors (46.9%), followed by T3 (41.7%) and T2 (11.4%). Most cases exhibited no nodal involvement (N0: 77.7%), whereas nodal metastasis was present in 22.3% of patients. Distant metastasis was rare (M1: 1.4%). Regarding TNM stage, 46.9% of patients were diagnosed at stage IV, 41.7% at stage III, and 11.4% at stage II. Tumor differentiation was predominantly moderate (G2: 64.4%), followed by poor (G3: 18.8%) and well-differentiated tumors (G1: 16.8%). Glottic tumors were the most common anatomical subtype (61.1%), followed by supraglottic (36%) and subglottic lesions (2.8%).

### 3.3. Treatment Characteristics and Patient Outcomes

Treatment modalities are presented in Table 3. All patients underwent surgical management, with total laryngectomy performed in 99.1% of cases. Cervical lymph node dissection was performed in 77.3% of patients. Concerning adjuvant therapy, 36% received radiotherapy alone, 27% chemoradiotherapy, and 1.4% chemotherapy alone, while 35.5% received no or unknown perioperative treatment. At the last follow-up, 34.6% of patients were alive and 65.4% had died. Among those who died, metastasis accounted for 48.6% of deaths, recurrence for 21.7%, and non–cancer-related causes for 27.5%.

### 3.4. Survival Analysis

**Figure 1:**
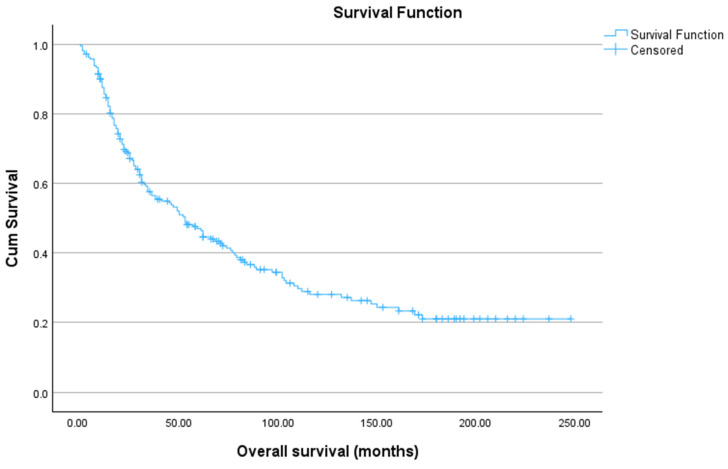
Overall survival of patients with laryngeal cancer during the period 1997–2017. The mean overall survival was 91.87 months [95% CI: 78.27–105.48]. Kaplan–Meier survival estimates were 87.6% at 1 year, 57.6% at 3 years, 47.0% at 5 years, and 28.1% at 10 years.

Kaplan–Meier analysis demonstrated a mean overall survival (OS) of 91.87 months (95% CI: 78.27–105.48) (Figure 1). OS rates at 1, 3, 5, and 10 years were 87.6%, 57.6%, 47.0%, and 28.1%, respectively.

**Figure 2:**
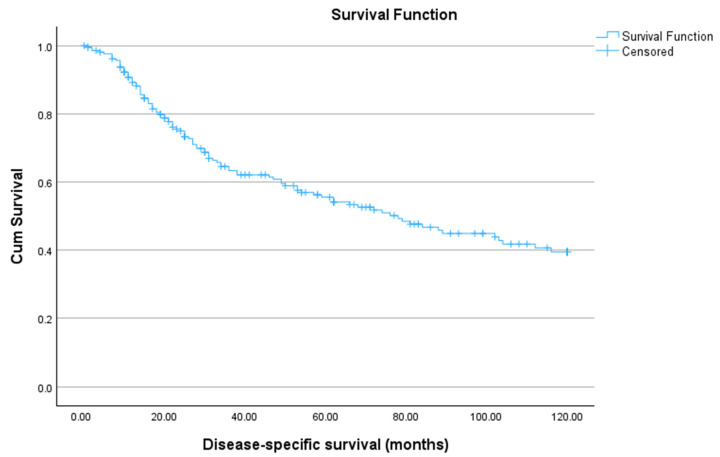
Disease-specific survival (DSS) of patients with laryngeal cancer during the period 1997–2017. The mean DSS was 72.06 months [95% CI: 65.27–78.85]. Kaplan–Meier estimates were 90.7% at 1 year, 64.5% at 3 years, 55.6% at 5 years, and 39.5% at 10 years.

Disease-specific survival (DSS) was 72.06 months (95% CI: 65.27–78.85), with 1-, 3-, 5-, and 10-year survival rates of 90.7%, 64.5%, 55.6%, and 39.5%, respectively (Figure 2).

**Figure 3:**
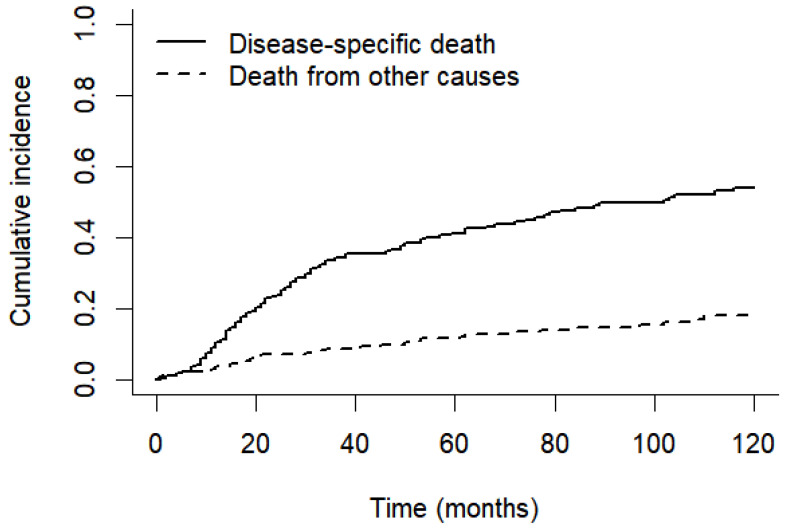
Cumulative incidence of disease-specific death and death from other causes over time.

Competing risks analysis showed that the cumulative incidence of disease-specific death was consistently higher than that of death from other causes throughout follow-up. At 120 months, the cumulative incidence of disease-specific death was approximately 0.55, whereas the cumulative incidence of death from other causes was approximately 0.18 (Figure 3).

Survival differed significantly across key clinicopathological variables (*p* < 0.001) (Table 4). More advanced TNM stages were associated with progressively worse outcomes, with stage II patients demonstrating the most favorable prognosis (5-year DSS: 76%), followed by stage III (66.7%) and stage IV (43.9%). A comparable gradient was observed for tumor size: patients with T2 tumors showed superior survival (5-year DSS: 69.6%) compared with those with T3 (62.6%) or T4 lesions (45.9%).

Nodal involvement exhibited the strongest prognostic effect. Patients with N0 disease achieved markedly better survival (5-year DSS: 62.2%) compared with those with nodal metastasis. N1 patients had extremely poor outcomes (5-year DSS: 16.7%; 10-year DSS: 0%), highlighting the aggressive biological behavior of node-positive disease. N2 and N3 patients also demonstrated substantially reduced long-term survival, with 5-year DSS rates of 45.9% and 21.4%, respectively. In addition, pre- and post-operative treatment modality was significantly associated with survival (*p* = 0.036). Patients receiving radiotherapy demonstrated comparatively higher 5-year survival rates, whereas those treated with chemoradiotherapy or chemotherapy alone exhibited lower survival, likely reflecting differences in disease severity and treatment selection (Table 4).

Conversely, age, tumor differentiation grade, anatomical subsite, and distant metastasis (M0 vs. M1) did not show statistically significant differences, although elderly patients (>80 years) and those with poorly differentiated tumors tended to exhibit numerically worse survival. Heavy alcohol consumption also showed a trend toward reduced survival but did not reach statistical significance in univariable analysis. Similarly, survival outcomes did not differ significantly between patients diagnosed in the earlier (1997–2006) and later (2007–2017) calendar periods (Table 4).

### 3.5. Multivariable Analysis

The multivariable Cox regression model (Table 5) identified tumor size (T) (HR = 1.665, *p* = 0.003) and nodal status (N) (HR = 1.546, *p* = 0.002) as independent predictors of disease-specific survival. Age, sex, distant metastasis, tumor location, differentiation grade, smoking exposure, alcohol use, and treatment modality were not independently associated with survival after adjustment. Multicollinearity among the covariates was evaluated using the variance inflation factor (VIF). In the final multivariable model, VIF values ranged from 1.05 to 1.30, indicating no evidence of multicollinearity. Higher VIF values were observed in preliminary models that included tumor size (T) (VIF: 5.06) and TNM stage (VIF: 5.72) simultaneously, reflecting moderate collinearity and supporting the decision to exclude TNM stage from the final model.

## 4. Discussion

This 21-year retrospective cohort study provides a comprehensive overview of the epidemiological, clinicopathological, and survival characteristics of patients with laryngeal squamous cell carcinoma (LSCC) in Western Greece. The predominance of male patients (95.3%) in our cohort is consistent with international data indicating that laryngeal cancer affects men disproportionately more often than women, reflecting historically higher rates of tobacco and alcohol use and greater occupational exposure to carcinogens among males [5,12,13]. Although women in our study exhibited a numerically higher mean survival than men, this difference did not reach statistical significance, likely due to the small number of female patients. Similar reports suggest that sex-related differences in survival may be influenced by biological factors, risk exposure patterns, and health-seeking behavior [14,15].

The mean age at diagnosis in our cohort (62.7 years) aligns with international observations that laryngeal cancer primarily affects middle-aged and older adults, with more than half of cases occurring in individuals aged ≥65 years [16]. The relatively narrow age distribution underscores the importance of targeted preventive strategies in this age group. In our survival analysis, patients aged 65–80 years showed the longest mean survival, whereas those >80 years had the poorest outcomes, highlighting the combined impact of advanced age, comorbidities, and limited tolerance to aggressive treatment [17,18]. These findings support the need for individualized treatment decisions that consider both chronological age and overall performance status.

Tobacco exposure emerged as a nearly universal risk factor in this population, with 97.6% of patients being smokers and a mean cumulative exposure of 88.15 pack-years. This level far exceeds the thresholds commonly associated with increased laryngeal cancer risk [19] and reinforces the well-established carcinogenic role of tobacco in head and neck malignancies [20,21]. Patients with ≤50 pack-years had higher long-term survival than those with >50 pack-years, although this difference did not reach statistical significance, possibly due to sample size, heterogeneity in smoking intensity and duration, or the cumulative impact of coexisting risk factors. Nevertheless, the observed trends are consistent with evidence linking heavy and prolonged tobacco exposure with poorer prognosis and increased competing mortality [19,21].

Alcohol consumption was also highly prevalent, with two-thirds of patients reporting moderate to heavy use. The combined effect of tobacco and alcohol is known to markedly amplify laryngeal cancer risk and may adversely affect treatment tolerance and overall health status [4,20,22,23,24]. In our cohort, non- or occasional drinkers had the best survival, followed by moderate drinkers, whereas heavy drinkers exhibited notably lower 5- and 10-year survival rates. Although these differences were not statistically significant in univariable analysis, alcohol use did not retain statistical significance in the multivariable Cox model. This suggests that its apparent effect may be influenced by confounding factors, including disease stage and overall tumor burden. Together with the extremely low rate of positive family history (0.9%), these data underscore the predominance of lifestyle and environmental determinants over hereditary factors in this setting and highlight the importance of integrated smoking and alcohol cessation interventions, ideally supported by structured counseling [15,22]. Dietary habits may also influence the risk of head and neck cancers; although nutritional patterns were not evaluated in the present study, previous research suggests that diets rich in fruits and vegetables may exert a protective effect [6].

The histopathological profile of our cohort reflects a substantial burden of advanced disease at presentation. Almost 90% of patients had stage III–IV tumors, and the majority presented with T3 or T4 lesions, indicating locally extensive disease. Only 11.4% had T2 tumors, underscoring the rarity of early-stage diagnosis in this region and echoing similar concerns raised in previous studies [25,26,27]. Survival analysis confirmed tumor size (T) as a strong prognostic factor: patients with T2 tumors achieved the highest survival rates, whereas those with T4 disease had markedly poorer outcomes, with significantly lower 3-, 5-, and 10-year survival. These observations are consistent with prior evidence linking larger primary tumor burden with increased risk of local invasion, treatment resistance, and disease-related mortality [27,28,29].

Although most patients had no nodal involvement at diagnosis (N0: 77.7%), nodal metastasis (N1–N3) was associated with substantially worse survival outcomes. In particular, patients with N1 disease exhibited the poorest long-term survival, including a 0% 10-year survival rate, emphasizing the aggressive biological behavior of node-positive tumors. Nodal status remained an independent predictor in multivariable analysis, associated with an approximately 1.55-fold increased risk of death, in agreement with published data identifying cervical lymph node metastasis as a pivotal determinant of prognosis, recurrence, and distant spread [30,31,32].

Distant metastases were uncommon at diagnosis (M1: 1.4%), reflecting the typical natural history of laryngeal cancer, where disease is often locally or regionally advanced at presentation but less frequently metastatic [33,34]. Nevertheless, patients with M1 disease had lower mean survival compared with M0 patients, despite the absence of statistically significant differences, likely due to the very small number of metastatic cases. Importantly, metastasis and locoregional recurrence accounted for the majority of cancer-related deaths in this cohort, underscoring the need for optimized systemic and locoregional control strategies, including contemporary systemic therapies such as immunotherapy and targeted agents [7,34,35].

TNM stage at diagnosis emerged as a key determinant of survival in univariable analysis. Patients with stage II disease had clearly favorable long-term outcomes compared with those with stage III and especially stage IV disease, with significant differences across survival curves. These findings are consistent with international data from large population-based cohorts, including SEER and Cancer Research UK, which report 5-year overall survival rates of approximately 60%, with substantially lower survival in advanced-stage disease, particularly stage IV (<40%) [10,16]. Similar findings have been reported in European studies, where stage III–IV disease is associated with significantly worse outcomes and increased mortality risk compared with early-stage tumors [25,36,37,38].

In our series, the 5- and 10-year OS rates were 47.0% and 28.1%, respectively, while the corresponding DSS rates were 55.6% and 39.5%. These findings are broadly comparable with those reported in similar populations with high proportions of advanced-stage tumors, although they highlight the persistent unmet need for earlier diagnosis and more effective treatment strategies. The discrepancy between OS and DSS may be partly explained by the proportion of deaths unrelated to laryngeal cancer. In our cohort, 38 of 211 patients died from other causes, indicating that non–disease-related mortality was not negligible and should be considered when interpreting survival outcomes. However, competing risks analysis demonstrated that disease-specific mortality remained consistently higher than mortality from other causes, supporting the validity of the DSS estimates.

Tumor differentiation was predominantly moderate (Grade 2), with smaller proportions of well- and poorly differentiated carcinomas. While patients with well-differentiated tumors (Grade 1) tended to have better long-term survival than those with poorly differentiated disease (Grade 3), these differences were not statistically significant. This pattern is in line with several reports indicating that histological grade, although biologically relevant, may not consistently retain independent prognostic value once stage and nodal status are taken into account [22,39,40].

Regarding anatomical subsite, glottic tumors were the most common, followed by supraglottic and rarely subglottic lesions, in agreement with national and international series [25,36,41,42,43,44]. Although glottic tumors are typically diagnosed earlier due to voice changes [3,45], survival in our cohort did not differ significantly by subsite, possibly reflecting the predominance of advanced-stage disease across locations and the limited sample size for subglottic cases.

Management in this cohort was predominantly surgical, with total laryngectomy performed in almost all patients, reflecting the advanced stage at diagnosis and the need for radical local control. Nearly 90% of patients presented with stage III–IV disease, and the majority had T3–T4 tumors, clinical scenarios in which total laryngectomy often remains an appropriate treatment strategy. In addition, the long study period (1997–2017) encompasses earlier years during which surgical management was more frequently employed before the widespread adoption of organ-preservation protocols based on chemoradiotherapy [32,34,46,47]. As a major tertiary referral center, our institution may receive a higher proportion of locally advanced cases requiring definitive surgical treatment. Furthermore, radiotherapy and chemoradiotherapy were widely used as adjuvant or perioperative treatments, whereas chemotherapy alone was uncommon, consistent with contemporary practice favoring multimodal regimens in locally advanced disease [7,26,34,48]. Pre- and post-operative treatment modality was significantly associated with survival in univariable analysis. Patients treated with radiotherapy demonstrated comparatively higher survival rates, whereas those receiving chemoradiotherapy or chemotherapy alone exhibited poorer outcomes. However, these findings should be interpreted with caution, as treatment allocation was not randomized and likely reflects underlying disease severity, with more intensive regimens being preferentially used in patients with more advanced or unfavorable disease characteristics [7,26,34,48].

In multivariable Cox regression analysis, tumor size (T) and nodal status (N) remained the only independent predictors of disease-specific survival. Larger tumors and nodal involvement were associated with increased mortality risk, confirming their central role in risk stratification and reinforcing the importance of early detection and appropriate management of locoregionally advanced disease [22,30,31,40]. The exclusion of TNM stage from the final multivariable model was based on its conceptual and statistical overlap with the individual T, N, and M components, which together capture the core elements of disease burden. Multicollinearity analysis using the variance inflation factor (VIF) demonstrated low values in the final model, confirming the absence of significant collinearity after model refinement. In preliminary models, higher VIF values were observed when TNM stage was included alongside its individual components, supporting the decision to simplify the model and improve interpretability [49].

Similarly, age, sex, tumor grade, anatomical subsite, pack-years, alcohol use, and treatment modality did not independently predict survival after adjustment, although they may influence outcomes in specific clinical contexts [22,39] and should still be considered in individualized decision-making.

This study has several notable strengths. It includes a relatively large and homogeneous cohort of patients with histologically confirmed laryngeal squamous cell carcinoma, managed over a 21-year period at a major tertiary referral center. The extended follow-up and evaluation of both overall and disease-specific survival at multiple time points offer a comprehensive overview of long-term outcomes. Moreover, the availability of detailed demographic, clinical, pathological, and treatment-related information enabled a robust analysis of prognostic factors using both Kaplan–Meier and multivariable Cox regression models. Importantly, this study provides much-needed epidemiological and survival data from Western Greece, a region underrepresented in the existing literature.

Nonetheless, certain limitations must be acknowledged. Although this study provides valuable insights into laryngeal cancer outcomes in Western Greece, the findings should be interpreted in the context of a single tertiary referral center. The University General Hospital of Patras serves as a major referral center for a large population across Western Greece, managing a wide spectrum of cases. However, as a tertiary care institution, it may also receive a higher proportion of advanced or complex cases, potentially introducing referral and selection bias. Therefore, while the findings reflect real-world clinical practice in this region, caution is warranted when generalizing the results to the broader population or to different healthcare settings. The retrospective, single-center design may limit external validity. Key variables, including HPV status, comorbidities, performance status, socioeconomic indicators, and detailed treatment parameters, were not consistently recorded and therefore could not be incorporated into the analysis. Smoking and alcohol data were self-reported, introducing potential recall bias. Some subgroups (e.g., women, subglottic tumors, and distant metastasis) were small, reducing statistical power. In addition, disease-specific survival (DSS) was calculated using standard censoring of non–cancer-related deaths; however, potential misclassification of the cause of death in retrospective data cannot be entirely excluded. Finally, the study spans more than two decades (1997–2017), during which diagnostic and therapeutic practices evolved and may have influenced patient management and survival outcomes.

## 5. Conclusions

This 21-year retrospective study provides important clinical and survival data on laryngeal cancer in Western Greece, highlighting a predominance of advanced-stage disease and a high burden of tobacco and alcohol exposure. Tumor size and nodal involvement emerged as the main independent predictors of survival, underscoring their central role in disease prognosis. Despite advances in treatment, long-term outcomes remain suboptimal, particularly among patients with node-positive or advanced tumors. These findings highlight the need for enhanced public health strategies, earlier symptom recognition, and optimized management pathways, while providing real-world evidence from a tertiary referral center to support targeted prevention and improved oncologic care.

## Figures and Tables

**Table 1:** Demographic characteristics of patients with laryngeal cancer (1997–2017).

Characteristic	Frequency (N)	Percentage (%)
Sex		
Male	201	95.3
Female	10	4.7
Total	211	100
Smoking status		
Smoker	206	97.6
Non-smoker	5	2.4
Total	211	100
Pack-years		
0–50 pack-years	47	22.3
51–100 pack-years	101	47.9
>100 pack-years	63	29.9
Total	211	100
Alcohol consumption		
Non-drinker	62	29.4
Occasional consumption	7	3.3
Moderate consumption	51	24.2
Heavy consumption	91	43.1
Total	211	100

**Table 2:** Histopathological characteristics of laryngeal cancer.

Characteristic	Frequency (N)	Percentage (%)
Tumor size (T)		
T2	24	11.4
T3	88	41.7
T4	99	46.9
Total	211	100
Nodal status (N)		
N0	164	77.7
N1	14	6.6
N2	26	12.3
N3	7	3.3
Total	211	100
Metastasis (M)		
M0	208	98.6
M1	3	1.4
Total	211	100
TNM stage		
Stage II	17	11.4
Stage III	81	41.7
Stage IV	113	46.9
Total	211	100
Tumor differentiation		
Grade 1 (Well-differentiated)	35	16.8
Grade 2 (Moderately differentiated)	134	64.4
Grade 3 (Poorly differentiated)	39	18.8
Total	208	100
Anatomical subsite		
Supraglottic	76	36.0
Glottic	129	61.1
Subglottic	6	2.8
Total	211	100
Family history		
Yes	2	0.9
No	209	99.1
Total	211	100

**Table 3:** Treatment modalities and clinical outcomes of patients with laryngeal cancer.

Variable	Frequency (N)	Percentage (%)
Pre-/Post-operative treatment		
None or unknown	75	35.5
Radiotherapy only	76	36.0
Chemotherapy only	3	1.4
Chemoradiotherapy	57	27.0
Total	211	100
Surgical treatment		
Yes	211	100
No	–	–
Total	211	100
Type of laryngectomy		
Total laryngectomy	209	99.1
Partial laryngectomy	2	0.9
Total	211	100
Vital status		
Alive	73	34.6
Deceased	138	65.4
Total	211	100
Cause of death		
Metastasis	67	48.6
Recurrence	30	21.7
Other, non–disease-related cause	38	27.5
Cancer cachexia	3	2.2
Total	138	100

**Table 4:** Survival rates at different time points according to prognostic factors (sex, age, tumor size, nodal status, metastasis, TNM stage, differentiation grade, pack-years, alcohol use, and treatment).

Prognostic Factor	1-Year Survival (%)	3-Year Survival (%)	5-Year Survival (%)	10-Year Survival (%)	Log Rank (χ^2^)	*p*-Value
Sex					0.261	0.609
Male	90.3	64.4	55	39		
Female	100	66.7	66.7	50.0		
Age range					3.143	0.370
<50	87	63.7	58.8	53.9		
50–64	92.7	64.3	56.4	36.2		
65–80	90.3	69.4	56.1	40.6		
>80	84.4	39.4	39.4	–		
Tumor size (T)					15.121	<0.001
T2	91.7	78.6	69.6	55.7		
T3	97.7	77.1	62.6	48.3		
T4	84	48.9	45.9	26.3		
Nodal status (N)					26.804	<0.001
N0	93.1	72.1	62.2	45.8		
N1	71.4	25	16.7	0.0		
N2	87.3	45.9	45.9	23		
N3	85.7	42.9	21.4	21.4		
Metastasis (M)					1.034	0.309
M0	91.6	65	55.8	39.5		
M1	33.3	33.3	33.3	33.3		
TNM stage					18.135	<0.001
Stage II	94.1	88.2	76	59.1		
Stage III	96.3	79.4	66.7	52.2		
Stage IV	86.1	49.5	43.9	25.8		
Tumor differentiation grade					3.444	0.179
G1 (Well differentiated)	94.2	65.6	62.2	54		
G2 (Moderately differentiated)	90.7	69.9	56.9	36.1		
G3 (Poorly differentiated)	87.1	46.3	46.3	41.7		
Pack-years					2.556	0.110
≤50	92.7	74.3	63.2	52.6		
>50	89.9	62.4	53.8	35		
Alcohol use					2.818	0.244
No or occasional	94	70.7	61.6	46		
Moderate	92	69.7	56.9	42.2		
Heavy	87.6	56.8	50.4	32.4		
Pre-/Post-operative treatment					8.544	0.036
None or unknown	91.5	67.4	54.5	47.3		
Radiotherapy	92.1	72.2	64.1	42.9		
Chemotherapy	66.7	33.3	–	–		
Chemoradiotherapy	89.1	51.2	46.3	18		
Calendar period					0.697	0.404
1997–2006	90.1	67	54.5	44.5		
2007–2017	91.3	61.5	56.9	20		

**Table 5:** Multivariable Cox regression analysis.

Variables in the Equation	B	SE	Wald	df	Sig.	Exp(B)	95% CI for Exp(B)	
							Lower	Upper
Sex	0.003	0.600	0.000	1	0.996	1.003	0.309	3.253
Age	0.021	0.012	2.926	1	0.087	1.021	0.997	1.046
Tumor size (T)	0.510	0.173	8.704	1	0.003	1.665	1.187	2.336
Nodal status (N)	0.435	0.139	9.765	1	0.002	1.546	1.176	2.031
Metastasis (M)	−1.041	0.820	1.609	1	0.205	0.353	0.071	1.764
Location	0.094	0.231	0.164	1	0.685	1.098	0.698	1.728
Tumor differentiation grade	−0.239	0.198	1.464	1	0.226	0.787	0.534	1.160
Pack years	0.000	0.003	0.027	1	0.870	1.000	0.994	1.005
Alcohol use	0.255	0.141	3.249	1	0.071	1.290	0.978	1.701
Pre-/Post-operative treatment	0.070	0.095	0.551	1	0.458	1.073	0.891	1.291

## Data Availability

The data presented in this study are available on reasonable request from the corresponding author. The data are not publicly available due to ethical and privacy restrictions.

## Abbreviations

The following abbreviations are used in this manuscript:

AJCC	American Joint Committee on Cancer
CI	Confidence Interval
DSS	Disease-Specific Survival
GDPR	General Data Protection Regulation
LSCC	Laryngeal Squamous Cell Carcinoma
OS	Overall Survival
TNM	Tumor–Node–Metastasis
